# Functionalized separator strategies accelerate the development of zinc-ion batteries

**DOI:** 10.1016/j.isci.2025.112787

**Published:** 2025-05-30

**Authors:** Yinghui Xue, Wanjiao Li, Runrun Liu, Yuepeng Lv, Jianxin Li, Dongdong Li, Yao Guo, Rui Hao, Huibing He

**Affiliations:** 1 School of Materials Science and Engineering, Anyang Institute of Technology, Anyang 455000, China; 2 School of Chemistry and Chemical Engineering, Guangxi Key Laboratory of Electrochemical Energy Materials, Guangxi University, Nanning 530004, China; 3 Henan Key Laboratory of Coal Green Conversion, Henan International Joint Laboratory of Coal Clean Utilization, College of Chemistry and Chemical Engineering, Henan Polytechnic University, Jiaozuo 454150, China

**Keywords:** energy engineering, energy systems, energy storage

## Abstract

Zinc-ion batteries have garnered significant attention owing to their high energy density, environmental sustainability, and cost-effectiveness. However, challenges such as zinc dendrite formation, hydrogen evolution, inert by-products, and zinc metal corrosion have substantially impeded the practical application of these batteries. Recently, functional modification of separators has emerged as a promising strategy to address these issues. This paper provides a comprehensive review of the research advancements in the functional modification of separators for zinc-ion batteries, elaborating on various modification approaches such as surface coating, composite material synthesis, and the development of hybrid structures. Furthermore, it elucidates the underlying mechanisms of these modifications and their applications in zinc-ion batteries. By critically analyzing the current technological limitations, this review proposes future development directions, such as exploring new materials and designing multi-functional separators, aiming to provide valuable guidance for advancing zinc-ion battery technology.

## Graphical abstract

**Figure:**
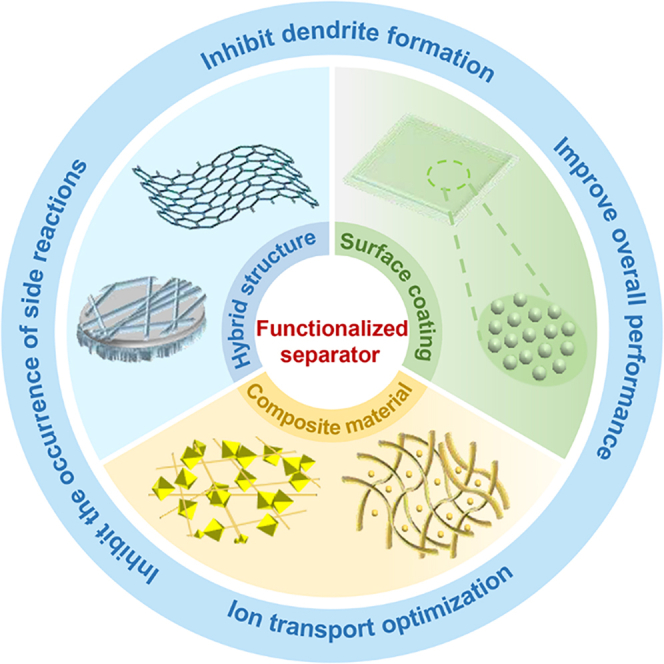

## Introduction

With the increasing global reliance on renewable energy, the demands placed on energy storage devices have become more stringent.^1^ Lithium-ion batteries (LIBs) have dominated the battery market owing to their advantages such as light weight and high energy density.^2^ However, when utilized in large-scale energy storage systems, LIBs encounter significant challenges in achieving high safety standards, cost efficiency, and minimizing environmental impact. The emergence of zinc-ion batteries (ZIBs) offers a promising resolution to these challenges. Compared with electrochemical devices such as lead-acid batteries and nickel-metal-hydride batteries, ZIBs have attracted much attention due to their superior characteristics, including higher energy density, better hydrogen evolution overpotential, enhanced safety, and cost-effectiveness. However, the practical application of ZIBs is constrained by several critical issues: notably, the low Coulombic efficiency (CE) and limited cycle life resulting from dendrite formation and side reactions (such as hydrogen evolution and corrosion) at the zinc metal anode.^3^^,^^4^^,^^5^ To address these challenges, a variety of strategies have been explored, leading to significant advancements. These strategies encompass regulating the zinc ion concentration gradient and homogenizing the zinc ion flux, inducing the formation of a protective layer on the zinc surface,^6^^,^^7^^,^^8^ and enhancing the electrochemical performance of ZIBs via rational design and optimization of the zinc anode structure.^9^^,^^10^^,^^11^^,^^12^^,^^13^

As a critical component of the battery, the separator is in direct contact with the anode, cathode, and electrolyte. It plays an essential role by ensuring electronic insulation between the anode and cathode while facilitating ion conduction when immersed in the electrolyte.^14^ However, traditional zinc-ion separators (such as filter paper and glass fiber [GF]) exhibit several limitations, including their inability to inhibit the growth of zinc dendrites and prevent side reactions, which significantly impact the battery’s service life. Consequently, numerous studies have been conducted in recent years to address these challenges, leading to substantial progress. Among these efforts, the functional modification of separators has emerged as a simple yet effective strategy. This approach enhances the electrochemical performance of both electrodes, thereby achieving high energy/power density and extended service life for ZIBs. By regulating the distribution of zinc ions within the separator and enhancing the kinetics of zinc ion migration, this method promotes stable and uniform zinc ion deposition, thus improving overall battery performance.^15^

**Figure 1:**
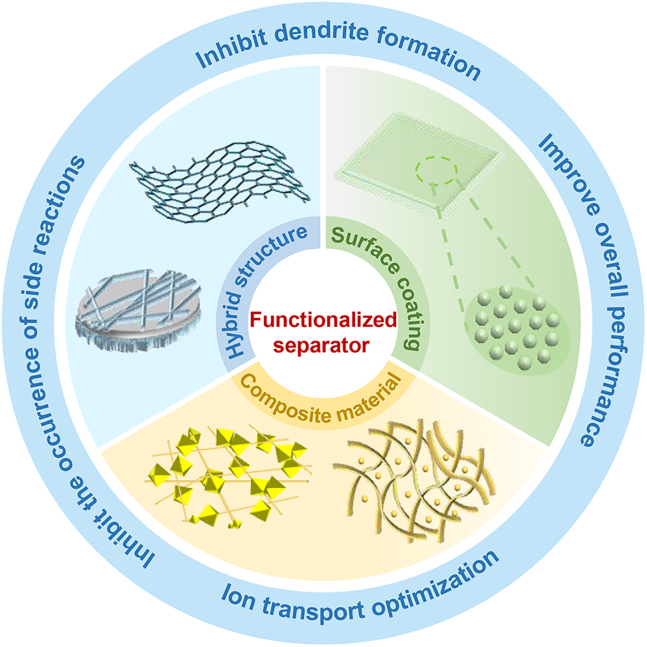
An overview of the strategies to modify the separators of ZIBs

Although several recent reviews have summarized the functionalities of separators for ZIBs,^16^^,^^17^^,^^18^ this study specifically addresses issues associated with the zinc anode and elaborates on corresponding improvement strategies (as illustrated in Figure 1). These strategies encompass the formation of surface coatings, the synthesis of composite materials, and the design of hybrid structures. Furthermore, the mechanisms underlying these improvements and their practical applications are thoroughly discussed. Lastly, this work examines the challenges in separator modification, such as complex fabrication processes and high costs, and investigates potential future development directions in the field of ZIBs.

## Strategies for functional modification of separators

### Surface engineering approaches

**Figure 2:**
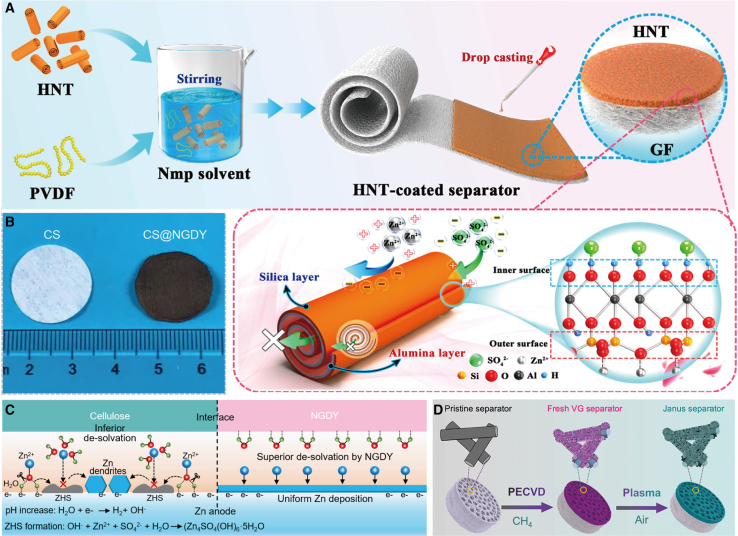
Schematic diagram of the preparation of the surface-coated separators (A) Schematic separator describing the preparation process of the HNT-GF separator and ion-sieving mechanism of HNT. (A) reproduced with permission from ref. 19 Copyright 2024 American Chemical Society. (B and C) Photographs of CS and CS@NGDY separators (B). Graphitic illustration of the stabilization of interfacial pH and the inhibition of unfavorable effects by using NGDY coating layer (C). (B and C) reproduced with permission from ref. 20 Copyright 2021 Wiley-VCH. (D) Schematic illustration of the fabrication process. (D) reproduced with permission from ref. 21 Copyright 2020 Wiley-VCH.

Surface coating represents an exceptionally effective approach in interface engineering. By employing surface coating techniques, the wettability of separators to electrolytes can be enhanced, thereby promoting the uniform deposition of zinc ions and reducing the likelihood of dendrite formation. Furthermore, batteries fabricated with coated separators exhibit superior stability and high-rate performance. Liu et al.^19^ successfully fabricated a functionalized double-sided separator by coating kaolinite nanotubes (HNTs) on GF. As depicted in Figure 2A, the differing electronegativities of the inner and outer surfaces of HNTs impart ion sieving characteristics to the HNT-GF separator, thereby significantly enhancing the migration number of zinc ions (t_Zn_^2+^ = 0.71). Simultaneously, the HNT-GF separator functions as an interfacial ion comb, regulating the flux of Zn^2+^, enabling multi-point progressive nucleation, reducing the nucleation overpotential, promoting more uniform deposition of Zn^2+^, and achieving dendrite-free and by-product-free deposition on the zinc anode. Yang et al.^20^ constructed a nitrogen-modified graphdiyne (NGDY) layer on the cellulose-polyester separator (CS) via a cross-coupling reaction. The surface of the obtained CS@NGDY separator appeared black (Figure 2B), contrasting with the white surface of the original CS separator, thereby confirming the successful modification with NGDY. Unlike traditional separators, the N atoms in the NGDY separator can capture electrons from water molecules in the solvation sheath, alleviating the coordination between H_2_O and Zn^2+^ and facilitating the desolvation of hydrated Zn^2+^ ions (Figure 2C).

Sun et al.^21^ developed a Janus separator by directly growing vertical graphene (VG) on one side of a commercial GF separator. As depicted in Figure 2D, this Janus separator was synthesized via a two-step method. Initially, plasma-enhanced chemical vapor deposition (PECVD) technology was utilized to grow VG nanosheets on the GF separator, using methane (CH_4_) as the carbon source. Subsequently, mild air plasma treatment was applied to the VG-coated separator, introducing additional oxygen- and nitrogen-containing functional groups onto the VG surface. The resultant 3D VG scaffold features a large surface area and porous structure, functioning effectively as an extension of the planar zinc anode. Consequently, the Janus separator achieves a uniform electric field distribution and mitigates the local current density at the anode/electrolyte interface while leveraging zincophilic properties to establish a consistent zinc ion flux. This innovative separator design enables the zinc-ion capacitor to achieve remarkable rate and cycling performance (retaining 93% capacity after 5,000 cycles at 5 Ag^−1^). Additionally, batteries incorporating this separator demonstrate exceptional energy density (182 Wh kg^−1^). Yao et al.^22^ fabricated the NPGO@GF separator by directly spraying polyaniline-modified graphene oxide (NPGO) onto one side of GF. The reversible transition between the protonated and deprotonated states of quinoline imide functional groups on NPGO nanosheets facilitates the rapid desolvation and transfer of Zn^2+^. Furthermore, metals such as tin (Sn) were deposited onto the surface of the cellulose separator via direct current magnetron sputtering to stabilize the zinc metal anode.^23^ Notably, alternative methods including drop casting and dip coating were also employed to achieve uniform surface coatings.^24^^,^^25^

The aforementioned separator modification involves a single-sided coating, which is exclusively applied to one side of the separator. Due to factors such as coating thickness and uniformity, this approach may result in non-uniform chemical and physical properties at the interface. Furthermore, during the charge-discharge process, variations in current density might cause one side of the coating to exhibit superior electrochemical performance while the other side remains constrained. In contrast, double-sided modification offers a more uniform and stable interfacial environment and can simultaneously regulate both the anode and cathode sides. For instance, Ma et al.^26^ proposed an ultra-thin and lightweight separator featuring an asymmetric functional coating. This coating can concurrently suppress the shuttling effect and continuous dissolution of transition metal ions on the cathode side and effectively inhibit dendrite formation, hydrogen evolution, and corrosion-related side reactions on the anode side. However, compared to single-sided coating, double-sided modification is associated with higher costs and greater process complexity.

### Composite material integration

#### Inorganic-organic hybrids

**Figure 3:**
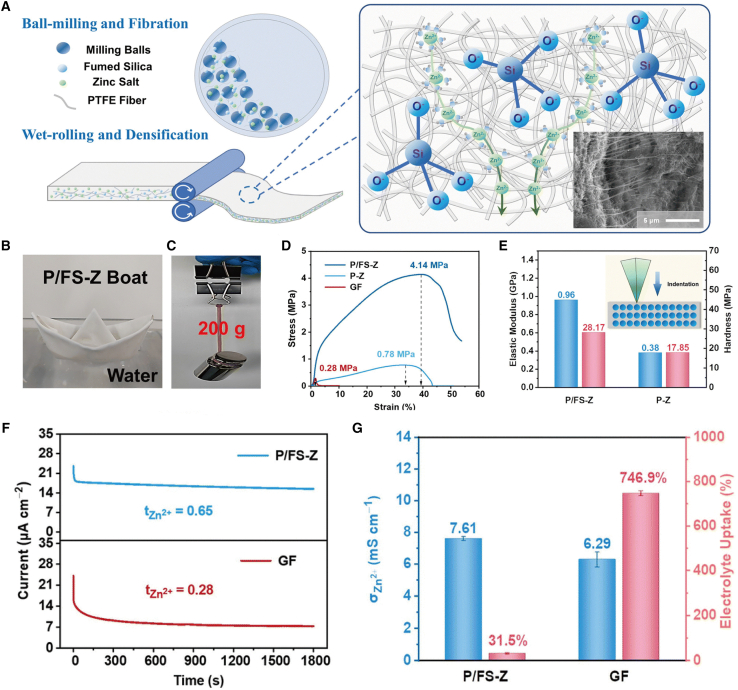
Schematic diagram of the preparation of organic-inorganic hybrid separators (A) Fabrication and structure schematic of the P/FS-Z separator (Inset: SEM image of the cross-section). (B) Demonstration of flexibility and hydrophilic-hydrophobic balance characteristics. (C) Demonstration of mechanical properties. (D and E) Tensile strength (D), elastic modulus (E), and hardness of P/FS-Z, P/Z, and GF. (F) CA tests of symmetric Zn||Zn cells with GF and P/FSZ separators. (G) Intrinsic conductivity and electrolyte uptake of P/FS-Z and GF separators. (A–G) reproduced with permission from ref. 27 Copyright 2023 Royal Society of chemistry.

In ZIBs, the modification of separators can be achieved through the construction of inorganic-organic composite materials. This approach enhances ion transport efficiency, mechanical strength, and interface stability and prolongs the cycle life of the battery. For example, Yao et al.^27^ developed a novel inorganic/organic hybrid separator fabricated from a hydrophobic polytetrafluoroethylene (PTFE) matrix, hydrophilic fumed silica (FS) filler, and zinc salt via a unique wet-rolling process (Figure 3A). The P/FS-Z separator integrates the high stability, high ionic conductivity, and strong chemical resistance of inorganic materials with the excellent flexibility of organic materials. The prepared P/FS-Z separator can be deformed into various shapes, such as a complex boat shape (Figure 3B), demonstrating its superior mechanical strength, outstanding flexibility, and significant potential for application in flexible wearable devices. As illustrated in Figure 3C, the P/FS-Z separator can support a weight of 200 grams, approximately 2,000 times its own weight. To further evaluate its mechanical properties, a tensile test was performed, and the results are presented in Figure 3D. The tensile strength of the P/FS-Z separator is 4.14 MPa, significantly higher than that of the P-Z separator without FS doping and the GF separator. Additionally, compared to the P-Z separator, the elastic modulus of the P/FS-Z separator increased markedly from 0.38 GPa to 0.96 GPa, and the hardness improved by 1.58 times (Figure 3E). These enhancements enable the P/FS-Z separator to effectively resist zinc dendrite formation and improve the cycle stability of the zinc anode in energy storage systems. According to the chronoamperometry (CA) test results, the P/FS-Z separator exhibits a higher Zn^2+^ transfer number (0.65 for P/FS-Z versus 0.28 for the GF separator) (Figure 3F). Furthermore, as depicted in Figure 3G, while the conductivity of the P/FS-Z separator is comparable to that of the GF separator, its lower electrolyte absorption rate confers an advantage in terms of energy density. More significantly, the newly developed P/FS-Z separator demonstrates an ultra-high cumulative zinc deposition capacity of up to 12,000 mAh/cm^2^ and achieves a record-breaking long-term cycling life of 700 h at a zinc discharge depth of 80%.

Wu et al.^28^ developed a cellulose/graphene oxide (Cellulose/GO, CG) composite separator. Cellulose is characterized by its low cost, satisfactory mechanical stability, and excellent chemical durability.^29^^,^^30^^,^^31^^,^^32^^,^^33^ On the other hand, GO exhibits superior wettability, stability, and a high specific surface area.^34^^,^^35^ Consequently, the combination of these two materials in the separator enables zinc deposition in a plate-like morphology rather than dendritic form, ensuring that the battery maintains exceptional reversibility at a low cost over thousands of cycles. Furthermore, the CG separator demonstrates excellent compatibility and flexibility with electrodes, as well as broad applicability when paired with diverse electrolytes, various cathode materials, and different battery configurations.

#### Conductive/functional additives

**Figure 4:**
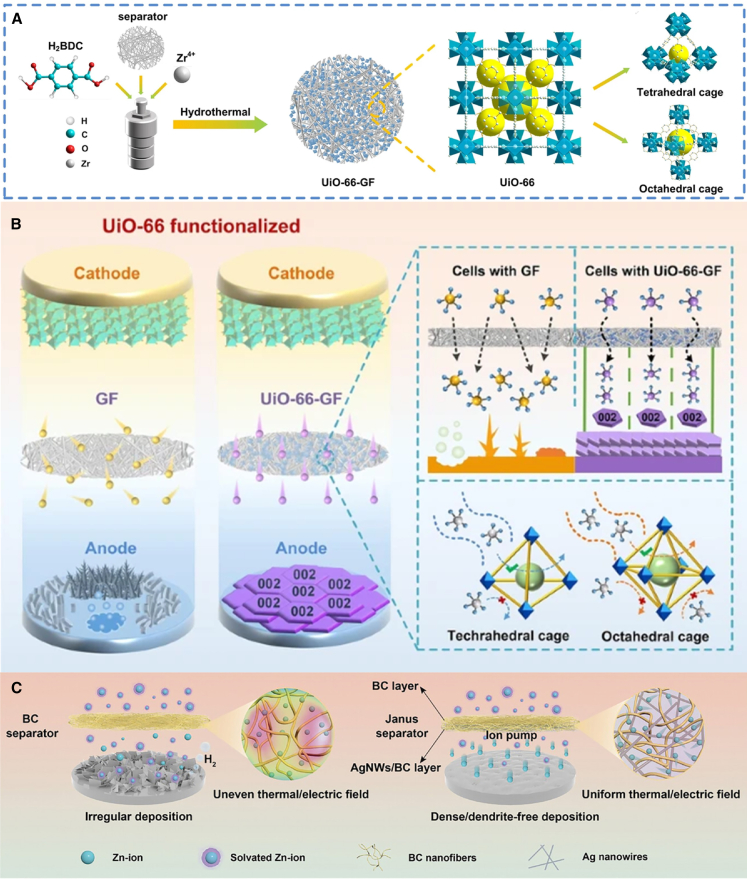
Schematic diagram of the preparation of composite separators (A and B) Preparation diagram of UiO-66-GF and structural diagram of UiO-66 (A). The deposition mechanism of GF and UIO-66-GF (B). (A and B) reproduced with permission from ref. 36 Copyright 2022 Springer Nature. (C) Schematic illustration of function mechanism of BC and Janus separators. (C) reproduced with permission from ref. 37 Copyright 2023 Wiley-VCH.

Adding functional materials to the separator of ZIBs can confer multiple advantages, including enhanced conductivity, improved mechanical strength, and increased battery stability. For instance, Song et al.^36^ fabricated a separator functionalized with Zr-based metal organic framework (UiO-66-GF) via the hydrothermal method for high-performance AZIBs (Figure 4A). The robust Zr-O bond coordination in UiO-66 enhances its stability under thermal, chemical, and aqueous conditions, which represents a significant advantage over other MOF materials. Additionally, the abundant Lewis acidic sites and porous channels within UiO-66 significantly improve ion transport capacity. As depicted in Figure 4B, compared to pristine GF, UiO-66-GF promotes the preferred orientation of zinc ions along the (002) crystal plane, thereby achieving uniform zinc deposition and effectively suppressing dendrite formation. Furthermore, density functional theory (DFT) calculations confirm that the (002) crystal plane exhibits a weak adsorption affinity for hydrogen atoms, which efficiently mitigates side reactions such as hydrogen evolution and corrosion, thus enhancing the battery’s corrosion resistance.

Cao et al.^37^ utilized bacterial cellulose (BC), which is characterized by abundant hydrophilic functional groups, green degradability, and non-toxicity, as the separator substrate. Subsequently, zincophilic silver nanowires (AgNWs) were incorporated to fabricate a cellulose-based double-sided heterogeneous Janus separator. This separator exhibits high mechanical strength, a uniform pore structure, uniform electric/thermal fields, and an ultra-thin profile. BC nanofibers, rich in hydroxyl groups, demonstrate excellent wettability with aqueous electrolytes (Figure 4C). Furthermore, silver, due to its strong affinity for zinc, facilitates efficient charge transfer with zinc ions and functions as an ion pump, enabling effective transport of Zn^2+^ through the electrolyte while guiding the uniform nucleation sites for subsequent zinc crystal growth.^38^^,^^39^^,^^40^ Consequently, symmetric cells based on the Janus separator can operate stably under ultra-high current densities (80 mA/cm^2^) and elevated temperatures (50°C). Moreover, the assembled AZICs and AZIBs full cells, as well as pouch cells, exhibit outstanding electrochemical performance. In another study, Cao et al.^41^ developed a carbon nanotube (CNT)-coated cotton towel separator (CT separator), enabling an ultra-long cycling life for the battery.

### Structural improvement

**Figure 5:**
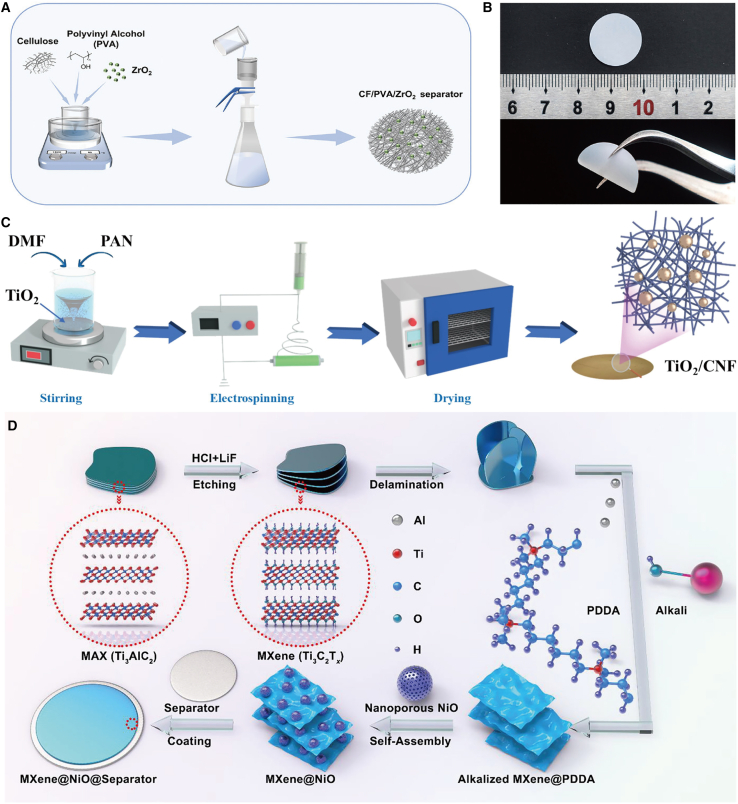
Schematic diagram for the preparation of the structure-improved separators (A and B) Schematic illustration of the fabrication of CF/PVA/ZrO_2_ separators (A). Digital photographs showing the appearance and flexibility of the CF/PVA/ZrO_2_ separator (B). (A and B) reproduced with permission from ref. 42 Copyright 2024 Wiley-VCH. (C) Preparation process of TiO_2_/CNF. (C) reproduced with permission from ref. 43 Copyright 2025 Elsevier Ltd. (D) Schematic illustration of synthesis process for the MXene@NiO modified separator. (D) reproduced with permission from ref. 44 Copyright 2022 American Chemical Society.

In addition to the aforementioned traditional modification strategies, advanced approaches such as asymmetric design, porous architecture, nanostructure engineering, and heterostructure construction of separators can also be employed to enhance the performance of ZIBs. For instance, Zhang et al.^42^ developed a cellulose/PVA/ZrO_2_ (CF/PVA/ZrO_2_) composite separator with an asymmetric structure (Figure 5A). One side features a ZrO_2_-rich functional layer designed to precisely control ion transport and deposition behavior, whereas the other side consists primarily of cellulose, which provides both mechanical support and ion-conducting capabilities. Furthermore, as illustrated in Figure 5B, the fabricated separator exhibits uniformity, flexibility, and potential applicability in flexible devices. The design philosophy of this separator is based on “mechanical-electrical synergy,” which effectively suppresses zinc dendrite growth through the integration of mechanical blocking and electric field regulation. Cellulose serves as a physical barrier to prevent dendrite penetration while maintaining excellent electrolyte wettability; ZrO_2_ nanoparticles induce uniform Zn^2+^ deposition via a polarized electric field, mitigating disordered dendrite growth caused by intensified local electric fields; PVA functions as a binder to reinforce the overall structural stability and strength of the separator. Experimental findings demonstrate that this innovative design substantially enhances the cycling performance and stability of the battery.

Ma et al.^26^ proposed an ultrathin and lightweight separator with an asymmetric functional coating, named “BPDA|PE|BisSF,” to achieve multi-scale interface stability and high energy density. On the side facing the positive electrode, biphenyl anhydride molecules (BPDA) were immobilized onto the polyethylene separator via spraying. The C=O and C-O-C functional groups in its structure can effectively coordinate with vanadium (V), thereby suppressing the shuttle effect and continuous dissolution of transition metal ions. Meanwhile, on the side facing the negative electrode, 3,3′-diamino-4,4′-dihydroxydiphenyl sulfone molecules were introduced to enhance the ion sieving capability of the separator. Specifically, the strong coordination interactions between the functional groups (-OH, -NH_2_, and -SO_2_) and Zn^2+^ ions promote a uniform Zn^2+^ flux and induce preferential growth along the (002) crystal plane. Additionally, the interaction with water molecules accelerates the desolvation process, effectively inhibiting dendrite formation, hydrogen evolution, and corrosion-related side reactions. Notably, a pouch battery with a capacity of 0.78 Ah fabricated using this separator achieved an excellent volumetric/mass energy density (133.3 Wh L^−1^/71.4 Wh kg^−1^) and an extremely high-power output (444.3 W L^−1^/238.0 W kg^−1^).

Furthermore, Liu et al.^43^ fabricated titanium dioxide fiber separators (TiO_2_/CNF) with a three-dimensional reticular structure via electrospinning technology (Figure 5C). The three-dimensional porous architecture of TiO_2_/CNF, in conjunction with the TiO_2_ modulation mechanism, facilitates a uniform Zn^2+^ ion flux and enhances ion transport kinetics, thereby promoting uniform zinc deposition and effectively mitigating side reactions. Feng et al.^44^ designed an independent, lightweight, zincophilic MXene/nanoporous NiO heterostructure-engineered separator. The preparation process is illustrated in Figure 5D: initially, MXene nanosheets are synthesized by etching the MAX phase. Subsequently, PDDA-alkalized MXene is obtained via a solution reaction conducted under argon protection. The MXene suspension is then mixed with nanoporous NiO to form the self-assembled MXene@NiO composite material. Finally, the homogeneous MXene@NiO suspension is sprayed onto the separator to fabricate the MXene@NiO-modified separator. The nanoporous NiO obtained through one-step vacuum distillation exhibits a large surface area, high porosity, and a uniformly porous structure, which can effectively homogenize the electric field distribution and reduce local current density. Moreover, the zincophilic property of the MXene@NiO layer facilitates a uniform zinc ion flux. Consequently, the designed separator ensures uniform Zn deposition, high reversibility, and suppression of side reactions.

**Figure 6:**
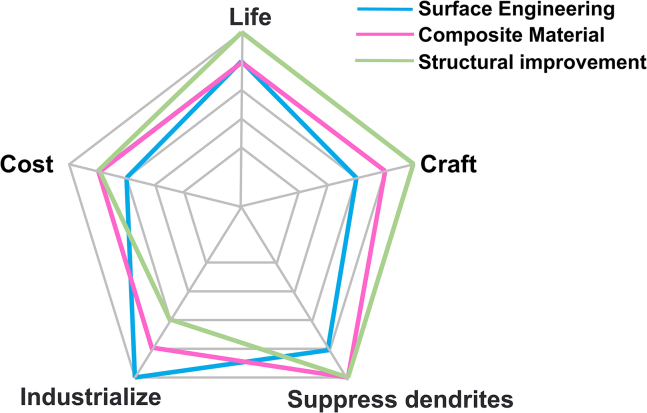
Comparison of different modification strategies in multiple aspects

As illustrated in Figure 6, the aforementioned three modification strategies exhibit distinct advantages and disadvantages across various dimensions. Among these, the surface engineering approach represents a straightforward and efficient diaphragm modification strategy, characterized by a simplified process and manageable costs. The composite material integration method leverages the synergistic benefits of multiple materials to achieve superior overall performance, making these two strategies the most promising for commercial application. Although the structural improvement strategy holds significant potential for long-term economic viability and performance breakthroughs, it remains largely theoretical due to its complex fabrication procedures and substantial capital investment requirements. In future research endeavors, it will be crucial to overcome these technical challenges in order to expedite the commercialization process and enhance both performance and efficiency within the broader battery system.

## Mechanisms and applications of functional modification

**Figure 7:**
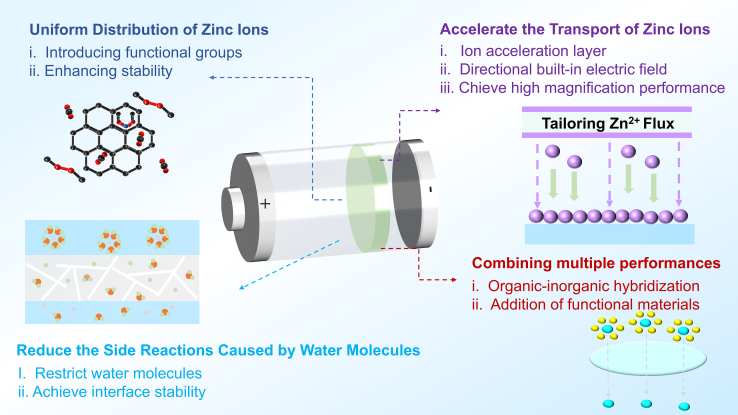
An overview of the application of functionalized separators

The functionalization of separators represents a critical trend in the advancement of secondary battery separators, particularly for ZIBs that utilize zinc metal as the anode. Given that zinc metal anodes are prone to side reactions such as dendritic growth and corrosion during electrochemical processes, stringent demands are placed on separator performance. Specifically, the separator must ensure uniform transport and deposition of zinc ions, suppress dendrite formation, and enhance battery stability. Additionally, it should reduce local current density, improve zinc ion transport efficiency for high-rate performance, and minimize side reactions to maintain interfacial stability (Figure 7). To date, researchers have employed diverse strategies to achieve separator functionalization, as detailed with specific examples in subsequent sections.

### Uniform distribution of zinc ions

**Figure 8:**
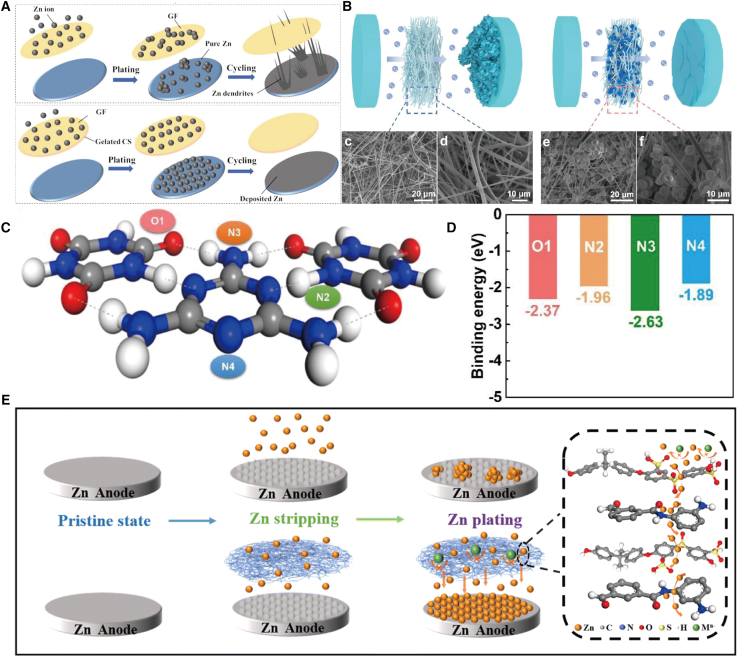
Schematic diagram of the preparation of a separator for suppressing zinc dendrites (A) The schematic illustrations for the functions of different separators (GF and GF@CS) during the Zn plating process. (A) reproduced with permission from ref. 45 Copyright 2023 Elsevier B.V. (B–D) Schematic illustrations of the blank and modified separator function during Zn plating/stripping process (B). Simulation of the interaction between Zn atoms and possible adsorption sites in the structure of supramolecules (C). The corresponding calculated binding energies (D). (B–D) reproduced with permission from ref. 46 Copyright 2021 Elsevier B.V. (E) The ion transport mechanism of SP separator in ZIBs. (E) reproduced with permission from ref. 47 Copyright 2022 Royal Society of Chemistry.

The non-uniform deposition of zinc ions on the zinc anode leads to the formation of zinc dendrites, which compromises the safety and stability of batteries and impedes the large-scale application of ZIBs. To mitigate the growth of zinc dendrites, functional groups such as amino and hydroxyl can be introduced to regulate the uniform deposition of zinc ions, thereby enhancing the stability of ZIBs. For instance, Yang et al.^45^ developed a zincophilic separator by coating and gelating natural chitosan (CS) onto one side of conventional GF separators (named CS-GF separator), which regulates zinc anode performance. As depicted in Figure 8A, the CS layer not only renders the surface of the GF separator smooth and uniformly distributed, facilitating the uniform transmission of zinc ions, but also the amino groups within CS possess the capability to adsorb zinc ions and guide their uniform distribution and transfer, thereby inhibiting dendritic growth during the zinc plating/stripping process. Additionally, the Zn symmetric cell employing the CS-GF separator exhibits remarkable long-term stability: 1,000 h at 2 mA cm^−2^/1 mA h cm^−2^; 500 h at 4 mA cm^−2^/1 mA h cm^−2^.

Liu et al.^46^ developed a unique functional supramolecular-modified separator (GF@SM). Owing to the superior filtering capability of GF@SM for randomly distributed zinc ions, uniform zinc deposition was achieved during the zinc plating-stripping process. Scanning electron microscope (SEM) analysis revealed that, compared with the pristine GF image, a significant number of supramolecules were observed growing on the microfibers in the GF@SM image (Figure 8B). Furthermore, DFT simulations demonstrated that carbonyl, triazine, imino, and amino groups exhibit strong interactions with zinc ions, promoting their orderly and dispersed distribution (Figures 8C and D), effectively suppressing the formation of dendritic zinc metal. In addition, constant current charge-discharge tests indicated that the symmetric battery employing the GF@SM separator could achieve an ultra-long lifespan of 2,000 h at a current density of 1 mA cm^−2^ while maintaining stable voltage polarization. The SPSF@PMIA (SP) separator devised by Hu et al.^47^ features -SO^3−^ groups that repel negative ions and negatively charged -CO-NH- groups that are electrostatically attracted to zinc ions, enabling selective passage of zinc ions and guiding their orderly deposition on the zinc anode surface, thus achieving a dendrite-free and stable zinc anode (Figure 8E). Chen et al.^48^ leveraged the dense and uniform nanopores of cotton cellulose, its abundant hydroxyl groups, excellent mechanical properties, and high ionic conductivity (56.95 mS cm^−1^). This approach reduces the zinc nucleation overpotential, accelerates zinc deposition kinetics, and effectively suppresses the formation of zinc dendrites and harmful side reactions.

### Accelerate the transport of zinc ions

**Figure 9:**
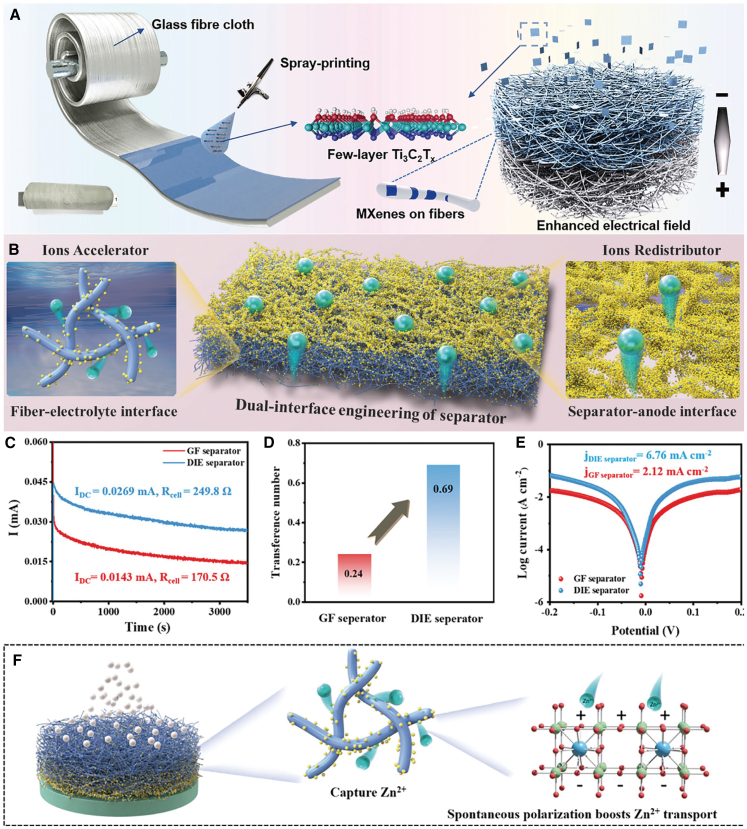
Schematic diagram of the preparation of the separator for optimizing ion transport (A) Schematic diagram depicting the preparation and function of MXene-GF separator. (A) reproduced with permission from ref. 49 Copyright 2022 Wiley-VCH. (B–F) The schematic diagram of dual-interface engineering of separator (B). Time-dependence response of potentiostatic DC polarization for Zn|Zn symmetric cells (C). The calculated transference number of zinc ions (t_Zn_^2+^) (D). Tafel plots of the Zn|Zn symmetric cells in the electrolyte of 2 M ZnSO_4_ at the scan rate of 1 mV s^−1^ (E). Schematic diagram of enhanced mechanism for Zn^2+^ transport via dual-interface engineering (F). (B–F) reproduced with permission from ref. 50 Copyright 2022 Wiley-VCH.

The high-rate performance of ZIBs is contingent upon the efficient transport of zinc ions. For instance, Su et al.^49^ developed a scalable Ti_3_C_2_T_x_ MXene-decorated Janus separator (Figure 9A). This innovative MXene-GF separator provides the Zn anode with two distinct advantages: first, the dielectric constant of the MXene-GF is substantially enhanced compared to that of bare GF, facilitating the establishment of a directional built-in electric field via the Maxwell-Wagner effect, thereby accelerating the migration of Zn^2+^. Second, the abundant surface functional groups of MXene ensure reduced desolvation energy, which not only expedites the diffusion of Zn^2+^ but also suppresses the anion flux. Therefore, dendrite-free zinc anodes can be realized in symmetric cells by employing MXene-GF, enabling stable cycling for 1,180 h at a current density of 1 mA cm^−2^ and 1,200 h at 5 mA cm^−2^. More remarkably, the full cell of the AZIBs assembled with the Janus MXene-GF separator exhibits excellent capacity retention, maintaining a retention rate of 77.9% after 1,000 cycles at a current rate of 5.0 A g^−1^.

Liang et al.^50^ introduced a distinctive dual interface engineering (DIE) strategy, wherein the separator was designed to function as an efficient ion transport regulator (Figure 9B). Potentiostatic direct current (DC) polarization tests were performed on zinc symmetric cells (Figure 9C). As shown in Figures 9D and E, following the DIE treatment of the separator, the Zn^2+^ transfer number of the symmetric cell increased from 0.24 to 0.69, whereas the exchange current density rose from 6.76 mA cm^−2^ to 21.2 mA cm^−2^, thereby enhancing the transport of Zn^2+^ at the electrode surface. Furthermore, from the standpoint of ion transport behavior, the enhancement mechanism based on the DIE-modified separator is elucidated in Figure 8F. The modification of BaTiO_3_ on the GF, along with the effective filling of surface gaps, not only facilitates the capture and acceleration of Zn^2+^ transport across the fiber-electrolyte interface but also ensures highly reversible Zn plating/stripping with ultra-high cumulative capacity. Xu et al.^51^ developed a COF-based separator (COF-Zn) with Zn^2+^ conductivity and electronegativity characteristics. The COF-Zn separator, featuring chemically grafted sulfonated zinc, selectively facilitates the transfer of Zn^2+^ ions, achieving high ionic conductivity (5.1 mS cm^−1^) and a high transfer number (0.87). Han et al.^52^ modulated the Zn^2+^ flux by incorporating an ion acceleration layer. ZnHCF, as a modifier, exhibits strong Zn^2+^ affinity and rapid diffusion channels, enabling efficient capture of Zn^2+^ on the electrode surface and swift transport to the deposition site. Consequently, the modified separator substantially enhances the cycle stability and rate performance of the full battery, thereby demonstrating its practical applicability and superiority.

### Reduce the side reactions caused by water molecules

**Figure 10:**
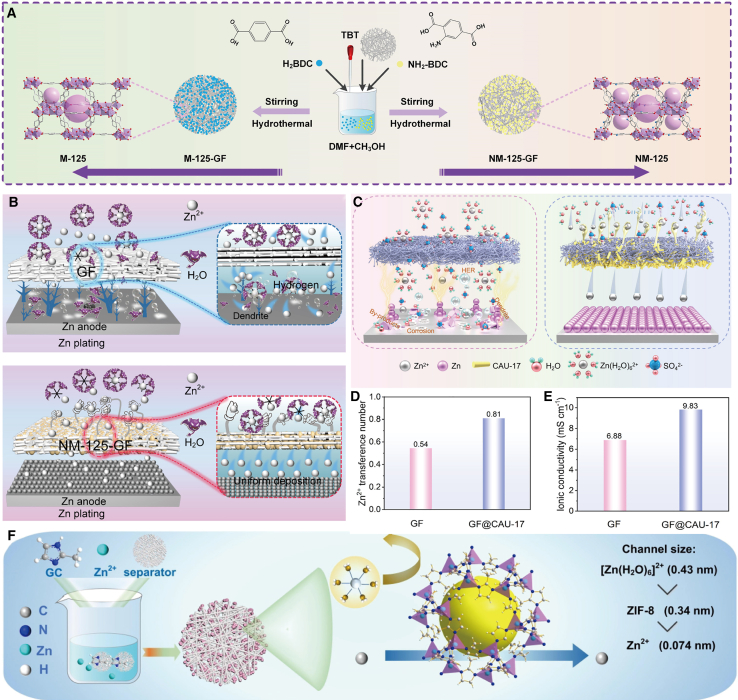
Schematic diagram of the preparation of the separators for suppressing the occurrence of side reactions (A and B) Preparation process of M-125-GF and NM-125-GF (A). Comparison of the mechanism of action of GF and NM-125-GF on zinc anode dendrites and side reactions (B). (A and B) reproduced with permission from ref. 53 Copyright 2024 Elsevier B.V. (C–E) Schematics of Zn deposition with GF (left) and GF@CAU-17 separators (right) (C). The Zn^2+^ transference number of the GF@CAU-17 and GF separators (D). The ionic conductivities of the GF@CAU-17 and GF separators (E). (C–E) reproduced with permission from ref. 54 Copyright 2024 Wiley-VCH. (F) Preparation process of ZIF-8-GF and desolvation process of ZIF-8. (F) reproduced with permission from ref. 55 Copyright 2023 Elsevier B.V. and Science Press.

In ZIBs, a meticulously designed and modified separator can effectively suppress side reactions while maintaining interfacial stability. For example, He et al.^53^ developed an MOF-functionalized separator. As illustrated in Figure 10A, compared with the conventional separator GF, the battery assembled with NM-125-GF exhibits a more uniform anode concentration field distribution and a flatter morphology after cycling. This separator effectively suppresses the formation of dendrites and side reactions by modulating the zinc ion flux (Figure 10B). Furthermore, the -NH_2_-functionalized M-125-GF displays certain zincophilic characteristics, which can reduce the solvation sheath layer of Zn^2+^ ions and suppress undesirable side reactions on the anode side. The battery assembled with this material also exhibits superior electrochemical performance. Specifically, the Zn|NM-125-GF|Zn battery can achieve stable cycling for up to 1,100 h at a current density of 0.5 mA cm^−2^. Additionally, the Zn|NM-125-GF|MnO_2_ battery showcases a high initial discharge specific capacity of 160.2 mAh g^−1^, with a capacity retention rate of approximately 99.8% after 700 charge-discharge cycles.

Hu et al.^54^ developed a GF separator coated with a bismuth-based metal-organic framework (CAU-17). This separator exhibits a unique zinc-ion self-recognition capability, enabling precise regulation of the ion transport process. As illustrated in Figure 10C, CAU-17, leveraging its exceptional zincophilic property, facilitates the desolvation of [Zn (H_2_O)^6^]^2+^, thus ensuring a uniform and rapid zinc-ion flux and achieving homogeneous zinc-ion deposition. Furthermore, CAU-17 is abundant in carboxyl functional groups, which effectively repel sulfate ions. Simultaneously, it disrupts the original hydrogen bond network of free water molecules and fundamentally inhibits side reactions and by-product formation by reconstructing hydrogen bonds. Additionally, to investigate the influence of CAU-17 on ion transport behavior, the ionic conductivity and Zn^2+^ transference number were evaluated. The results, as shown in Figures 10D and E, indicate that the GF@CAU-17 separator exhibits a higher Zn^2+^ transference number (0.81) and ionic conductivity (9.83 mS cm^−1^). The CAU-17-modified separator demonstrates remarkable stability due to its distinctive performance advantages.

Zhang et al.^55^ fabricated a ZIF-8-GF separator with uniformly distributed pores through *in situ* growth (Figure 10F). This separator possesses advantages such as a high specific surface area, excellent hydrophobicity, and abundant metal sites. Moreover, the hydrophobic nature of ZIF-8 effectively prevents active water molecules in the electrolyte from reaching the zinc metal anode interface, thus suppressing harmful side reactions including corrosion and hydrogen evolution. Ge et al.^56^ employed a single-ion Zn^2+^ conductive nanocellulose separator as the separator. This functional separator alleviates issues related to zinc anode corrosion and hydrogen evolution reaction by synergistically optimizing critical properties such as mechanical strength, preferred Zn^2+^ conductivity, and hydrophilicity, achieving stable cycling with a depth of discharge exceeding 80% under high areal capacity conditions (8.0 mAh cm^−2^).

### Combining multiple performances

**Figure 11:**
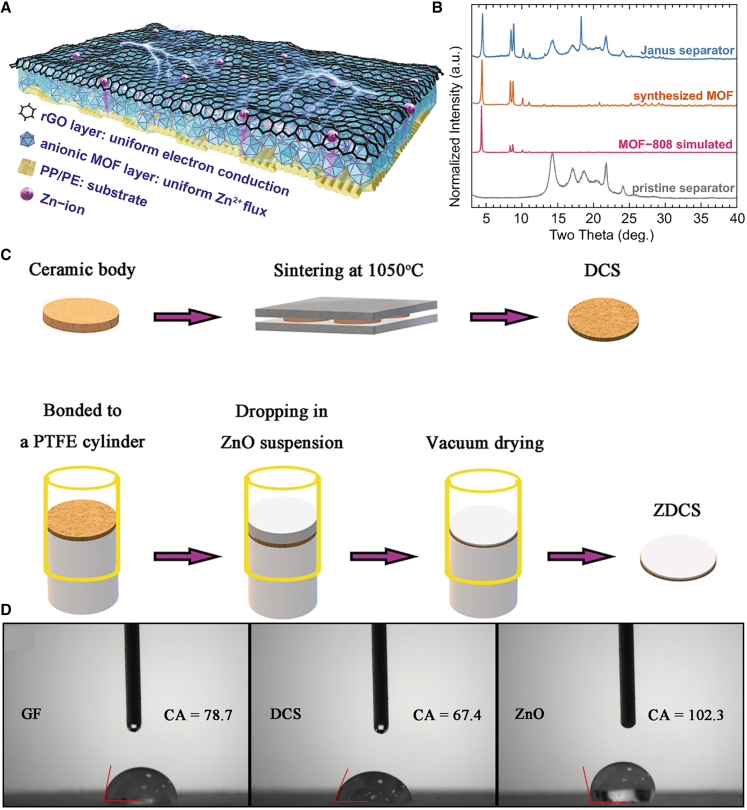
Schematic diagram of the preparation of multi-functional integrated separators (A and B) Schematic illustration for the Janus separator (A). XRD patterns of the simulated MOF-808, synthesized MOF, pristine separator, and Janus separator (B). (A and B) reproduced with permission from ref. 57 Copyright 2021 Springer Nature. (C and D) Schematic diagram of preparing DCS and ZDCS (C). Contact angle between materials and electrolyte for GF, DCS, and ZnO (D). (C and D) reproduced with permission from ref. 58 Copyright 2024 Elsevier Ltd.

Multifunctional integration pertains to the design and fabrication process of the separator in ZIBs, where multiple properties and functions are integrated to achieve comprehensive optimization of the separator material. The primary objective is to enhance various critical performances of the separator, thereby enabling more effective support for overall battery performance enhancement in practical applications. For instance, Pan’s^57^ group developed a Janus separator by integrating zinc-ion conductive metal-organic frameworks (MOFs) with reduced graphene oxide (rGO) (Figures 11A and B). This design enables simultaneous regulation of the uniform Zn^2+^ flux and electron conduction during battery operation. The incorporation of the MOF/rGO bifunctional interlayer enhances the stability of the zinc anode, as evidenced by its stable plating/stripping behavior (lasting over 500 h at 1 mA cm^−2^), high coulombic efficiency (99.2% after 100 cycles at 2 mA cm^−2^), and a significantly reduced redox potential barrier.

Yang et al.^58^ coated the surface of a diatomite ceramic separator with nano-scale ZnO to construct a three-dimensional porous structure (as shown in Figure 11C). To evaluate the wettability of the material with the electrolyte, GF fibers, DCS powder, and ZnO powder were compacted to eliminate the influence of pore structure on the test, and the contact angles between the materials and the electrolyte were measured. The results are presented in Figure 11D. Compared with GF fibers, the compacted DCS powder exhibits a smaller contact angle with the electrolyte, indicating superior wettability of the DCS material compared to GF. ZnO powder demonstrates significant hydrophobicity. Consequently, the ZnO coating on the ZDCS surface effectively minimizes the contact between H_2_O in the electrolyte and the Zn anode, suppressing side reactions between H_2_O and the Zn anode. This combination ensures more uniform zinc ion deposition on the Zn anode surface, effectively inhibiting dendrite growth and by-product formation. Furthermore, the battery assembled with the ZnO-coated diatomite ceramic separator exhibits enhanced specific discharge capacity and improved capacity retention.

Wei et al.^59^ utilized zwitterionic surfactant SBMA to fabricate a modified PAN separator. The PAN@SBMA separator, prepared via electrospinning, exhibits excellent flexibility and optimal surface morphology characterized by uniform fiber diameter and pore size distribution. As a result, Zn symmetric cells with PAN@SBMA separators show long-term stability (up to 1700 h), which outdistances the cell with GF-D (only up to 50 h) under current density at 1 mA cm^−2^. Meanwhile, the Zn//PAN@SBMA//NH4V4O10 full cells have high specific capacity (236 mAh g^−1^) and excellent long-term durability with 84.2% capacity retention after 2,000 cycles at 5 A g^−1^.

## Challenges and future perspectives

### Current limitations

The fabrication and modification of functionalized separators typically involve multi-step synthesis and recombination, which encompass intricate technological processes. Furthermore, high-performance separator materials are often associated with high costs, thereby escalating production challenges and energy consumption. Additionally, research on separators for ZIBs remains predominantly at the laboratory stage. While existing modified separator materials exhibit satisfactory performance in small-scale laboratory settings, they may encounter significant issues such as consistency, stability, and quality control during large-scale manufacturing, thus severely restricting their widespread application.

### Emerging opportunities

#### Smart separators

In the future, it is anticipated that temperature-sensitive, Ph-sensitive, or electrochemically active materials will be integrated into separators to enable real-time regulation of pore structure or surface charge. Additionally, incorporating self-healing materials into separators is expected to extend their service life by automatically repairing microcracks generated during cycling processes. For instance, Fan et al.^60^ developed a room temperature self-healing supramolecular composite material composed of polyurethane (PU) elastomer, cellulose nanocrystals (CNC), and multiple dynamic bonds. In this system, the abundant hydroxyl groups on the CNC surface form numerous hydrogen bonds with the PU elastomer, creating a dynamic physical crosslinking network. This network facilitates self-healing without compromising mechanical properties. The results demonstrate that the obtained supramolecular composite material exhibits high tensile strength (24.5 ± 2.3 MPa), excellent elongation at break (1484.8 ± 74.9%), remarkable toughness (156.4 ± 31.1 MJ m^−3^, equivalent to spider silk and approximately 5.1 times higher than aluminum), and outstanding self-healing efficiency (95 ± 1.9%).

#### AI-driven material discovery

**Figure 12:**
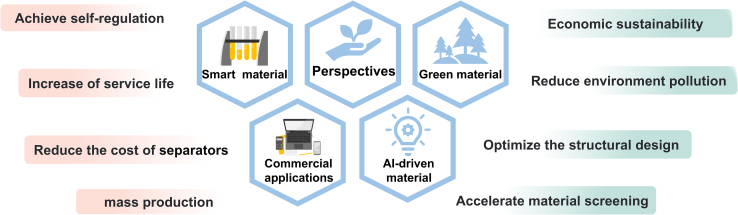
A schematic diaphragm of the research direction outlook for zinc separators modification

Artificial intelligence (AI) technology can substantially reduce the research and development cycle of separators by accelerating material screening, optimizing structural design, and predicting battery behavior. Furthermore, high-throughput material screening enabled by AI allows for rapid prediction of key properties such as ionic conductivity, mechanical strength, and chemical stability of candidate materials. Additionally, AI-driven approaches facilitate multi-objective performance optimization. For instance, Wang et al.^61^ developed an AI-powered automated materials laboratory named “Polybot.” Using poly (3,4-ethylenedioxythiophene) doped with poly (4-styrenesulfonic acid) (PEDOT: PSS) as a case study, the researchers successfully devised a manufacturing process for a transparent conductive film via Polybot’s automated experiments, achieving an average conductivity exceeding 4,500 S/cm. Through the Polybot platform, researchers can efficiently navigate the complex parameter space and rapidly identify optimized processing conditions. This approach not only enhances experimental efficiency but also minimizes the impact of human bias on experimental outcomes. In the future, this AI-assisted automated technology is anticipated to be applied across a broader range of material systems, potentially leading to significant advancements in materials science (Figure 12).

#### Green economy separators

The implementation of green economy zinc-ion battery separators offers numerous advantages, particularly in the realms of environmental protection and economic sustainability. First, these separators typically utilize renewable and eco-friendly materials, thereby decreasing reliance on conventional resources during production, as well as reducing carbon emissions and minimizing environmental pollution. Furthermore, through the judicious selection of materials and innovative separator design, the energy density and cycle life of the battery can be enhanced, ensuring consistent high performance over extended periods of use. Consequently, this approach provides a more efficient energy storage solution while simultaneously lowering costs.

Yang et al.^62^ developed an economical, efficient, and multifunctional separator composed of industrial waste fly ash particles and cellulose nanofibers, fabricated via a straightforward solution-coating method. The resultant fly ash-cellulose (FACNF) separator exhibits high ionic conductivity (5.76 mS/cm) and a low desolvation energy barrier for hydrated zinc ions, which facilitates rapid ion transport kinetics and suppresses water-induced side reactions. Furthermore, symmetric cells and full cells incorporating the FACNF separator demonstrate prolonged and stable cycling performance without dendrite formation on the electrode surface. Additionally, a pouch cell equipped with the FACNF separator can reliably power a digital thermometer for up to 2 months, with no gas generation or volume expansion during this period. Qin et al.^63^ synthesized a novel degradable separator (ZnHAP/BC) that restricts water molecule activity and enhances ion transport kinetics by integrating natural and eco-friendly bacterial cellulose (BC) with nano-hydroxyapatite (HAP), achieving a stable, reversible, and highly utilized zinc metal anode. Cao et al.^64^ fabricated a cost-effective CNF+LMS separator by blending cellulose nanofibers (CNF) with lithium magnesium silicate (LMS). This separator possesses superior wettability, significant tensile strength, and outstanding ionic conductivity. Notably, the cost of the CNF+LMS separator is as low as 6.36 yuan per square meter, significantly lower than that of currently available commercial and reported separators.

## Conclusion

The separator is an essential component of ZIBs and plays a critical role in ensuring their normal operation. An ideal separator should possess characteristics such as thinness, excellent thermal stability, sufficient mechanical strength, appropriate pore structure, and superior electrochemical performance. These attributes contribute to the development of ZIBs with high safety and outstanding performance. In this paper, we systematically summarize strategies for functional modification of separators in ZIBs, elucidate the underlying mechanisms of these modifications, discuss their relevant applications, and a list of abbreviation (Table 1) is attached at the end of the article. Furthermore, based on a comprehensive analysis of recent advancements in separator technology, we highlight the challenges and future prospects of separator modification for ZIBs. Currently, research on separators for ZIBs remains largely confined to laboratory settings. High-performance separator materials often entail significant costs, thereby increasing production complexity and energy consumption, which severely restricts their large-scale application. For the future commercialization of ZIBs, this paper proposes strategies such as the development of smart separators and green economical separators to guide the future direction of ZIB separator research.

**Table 1:** List of abbreviations

Abbreviation	Definition	Abbreviation	Definition
LIBs	lithium-ion batteries	VG	vertical graphene
ZIBs	zinc-ion batteries	PECVD	plasma-enhanced chemical vapor deposition
CE	Coulombic efficiency	CH4	methane
GF	glass fiber	NPGO	polyaniline-modified graphene oxide
HNTs	kaolinite nanotubes	PTFE	polytetrafluoroethylene
NGDY	nitrogen-modified graphdiyne	FS	fumed silica
CS	cellulose-polyester separator	CA	chronoamperometry
CG	cellulose/graphene oxide	UiO-66-GF	Zr-based Metal organic Framework
AZIBs	aqueous Zinc-Ion Batteries	DFT	density Functional Theory
AZICs	aqueous Zinc-Ion Capacitor	CNT	carbon nanotube
BC	bacterial cellulose	AgNWs	silver nanowires
CT	cotton towel	PVA	polyvinyl Alcohol
BPDA	biphenyl anhydride molecules	CNF	carbon Nanofiber
PDDA	poly dimethyl diallyl ammonium chloride	SEM	scanning electron microscope
PMIA	m-phenylene isophthalamide	DIE	dual interface engineering
DC	direct current	COF	covalent Organic Frameworks
ZnHCF	zinc hexacyanoferrate	NM-125	-NH_2_-functionalized material
rGO	reduced graphene oxide	PAN	polyacrylonitrile
SBMA	sulfobetaine methacrylate	PU	polyurethane elastomer
CAU-17	bismuth-based metal-organic framework	ZIF-8	zeolitic Imidazolate Frameworks
CNC	cellulose nanocrystals	AI	artificial intelligence
FACNF	fly ash-cellulose	LMS	lithium magnesium silicate

Smart separators with self-identification capabilities can selectively enhance the migration of zinc ions and dynamically adjust their structure in response to the operational state of the battery, thereby comprehensively improving the electrochemical performance of ZIBs. By leveraging AI models to analyze performance data and predict the influence of various materials and designs on battery performance, the selection of novel materials and the optimization of separator structures can be effectively guided. Additionally, natural materials (e.g., cellulose, chitosan, etc.) can serve as the basis for separator fabrication, enhancing the environmental sustainability of the production process. Looking ahead, overcoming challenges related to large-scale manufacturing, deepening fundamental mechanistic studies, and exploring innovative approaches that integrate intelligent systems and multi-disciplinary technologies will be pivotal in advancing the commercialization of ZIBs.

## Acknowledgments

This work was supported by the 10.13039/501100004607Natural Science Foundation of Guangxi Province (2024GXNSFAA010271), the 10.13039/501100001809National Natural Science Foundation of China (22308066), the Academic Newcomer Award Project of Guangxi University (2025GXUXSXR07), the Science and Technology Project of Henan Province (No. 242102240085), the Postgraduate Education Reform and Quality Improvement Project of Henan Province (No. YJS2023JD60), the 10.13039/501100008528Anyang Institute of Technology, Anyang, Henan, Specialized Research Fund for the Doctoral (No. BSJ2021022), the Key Research Project of Henan Provincial Higher Education (No. 24A430001 and 22B140001), the Science and Technology Project of Anyang (No. 2023C01GX016), the 10.13039/501100006407Natural Science Foundation of Henan Province (No. 252300420018), and Anyang Institute of Technology energy power a new round university-level key discipline.

## Author contributions

Y.H.X. and H.B.H. conceived the idea and designed the frame. W.J.L. wrote the first draft of the manuscript. All authors commented, edited, and revised the final manuscript.

## Declaration of interests

The authors declare no conflicts of interest.
